# Differentiation of three common deep-water hermit crabs (Crustacea, Decapoda, Anomura, Parapaguridae) from the South African demersal abundance surveys, including the description of a new species of *Paragiopagurus* Lemaitre, 1996

**DOI:** 10.3897/zookeys.676.12987

**Published:** 2017-05-23

**Authors:** Jannes Landschoff, Rafael Lemaitre

**Affiliations:** 1 Department of Biological Sciences and Marine Research Institute, University of Cape Town, Rondebosch 7701, South Africa; 2 Department of Invertebrate Zoology, National Museum of Natural History, Smithsonian Institution, 4210 Silver Hill Road, Suitland, MD 20746, U.S.A.

**Keywords:** Crustacea, Parapaguridae, *Paragiopagurus*, new species, hake, *Merluccius* spp., South Africa

## Abstract

Deep-water hermit crabs of the family Parapaguridae can be abundant (up to 20 kg or 1000 hermit crab individuals per haul) in the trawl bycatch collected during South African demersal abundance research surveys. Until recently, only two parapagurid species had been recognized in the bycatch; *Parapagurus bouvieri* Stebbing, 1910, and *Sympagurus dimorphus* (Studer, 1883). Detailed examination of numerous samples of parapagurid specimens from research surveys revealed the existence of a third, undescribed species previously confounded with *S. dimorphus*, but in fact belonging to a different genus. This new species, *Paragiopagurus atkinsonae* **sp. n.** is the 25^th^ in the genus *Paragiopagurus* Lemaitre, 1996, and has been found only in a small region on the West Coast shelf of South Africa, at depths of 199–277 m. The species is herein fully described and illustrated, including colour images, µCT scans of selected body parts, and CO1 barcode data. The new species is morphologically most similar to *P. ventilatus* Lemaitre, 2004, a species associated with hydrothermal vents, but differs in armature of the fourth antennal segment (armed with a spine on the dorsolateral distal angle vs. unarmed in *P. ventilatus*); setation of the antennal flagella (nearly naked vs. with dense setae in *P. ventilatus*); plumose setation on the third maxillipeds and basal segments of chelipeds (absent vs. present in *P. ventilatus*); number of rows of scales on the propodal rasp of pereopod 4 (two or three rows vs. one row in *P. ventilatus*); and degree of telson asymmetry (weakly asymmetrical vs. strongly asymmetrical in *P. ventilatus*). *Paragiopagurus atkinsonae* **sp. n.** is superficially similar to *S. dimorphus*, with males of the two species showing the same extreme degree of sexual dimorphism on the right cheliped, general light orange colouration, and frequent use of colonial zoanthid carcinoecia for pleonal protection. To aid in future identifications and to facilitate data gathering during surveys, a comparison of *P. atkinsonae* **sp. n.** with *S. dimorphus* is provided, along with descriptions of colouration and photographs of live specimens of all three parapagurid species. Information on taxonomy of the species is summarized, as well as knowledge of their distribution in the demersal research survey regions of South Africa.

## Introduction

**Figure 1.**
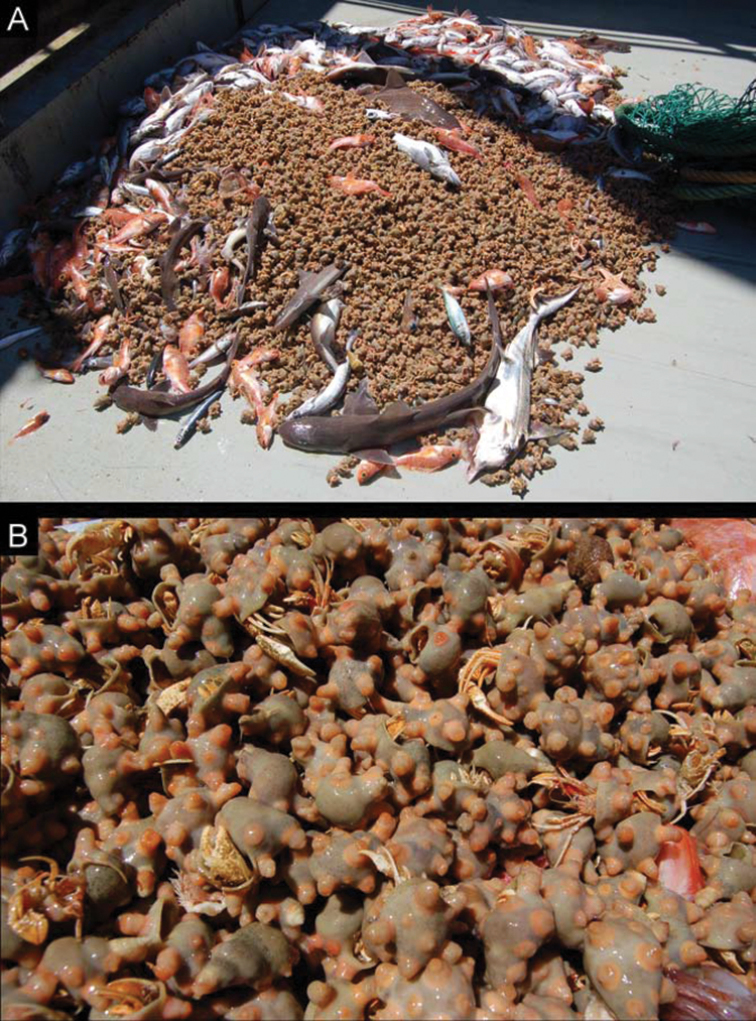
Abundance of deep-water hermit crabs in South African demersal research survey, Agulhas Bank, South Africa, Nan2007 401, sta 1294–008, S35°24.40', E19°10.70', 227 m, 12 Jan 2007: **A** contents of one trawl showing catch **B** close-up of parapagurid specimens and anthozoan symbionts (colonies of *Epizoanthus* sp.) in same. (Photographs by Kerry Sink).

The South African Department of Agriculture, Forestry and Fisheries (DAFF, formerly Department of Environmental Affairs and Tourism) has conducted biannual demersal fishery surveys since 1986. To assess the stock status of commercial fish species such as South African hake (*Merluccius* spp.), two ‘demersal surveys’ are usually conducted every austral summer (West Coast) or autumn (South Coast). In some years, the two surveys are repeated during the winter or spring. Each survey conducts between 100–120 trawls, the majority of these take place between the 100–500 m isobaths, but some trawls extend to depths >1000 m (Yemane et al. 2009). Among the invertebrate bycatch retained in research trawls, deep-water hermit crabs of the family Parapaguridae are particularly common and occasionally, remarkably abundant, although they have not always been adequately monitored. On the West Coast, a trawl can contain up to 20 kg (about 1000 individuals) of parapagurids per haul, and these can make up the vast majority of all invertebrates retained in the research trawls (Fig. 1; L. Atkinson, pers. comm.). Such parapagurid abundance is an indication of their ecological importance on the South African continental shelf. The exact role, however, that these anomuran crustaceans might play in the benthic community remains to be studied.

Since 2011, invertebrate bycatch, including parapagurids, have been monitored more consistently in research surveys, as part of DAFF’s commitment to developing an ecosystem approach to management. Based on limited benthic taxonomic literature from the region, biologists identified only two abundant parapagurid species, *Sympagurus dimorphus* (Studer, 1883) and *Parapagurus bouvieri* Stebbing, 1910. However, during the January 2012 West Coast survey on the RS Africana, an unfamiliar male parapagurid specimen with “green eyes” was noticed and collected by Dr. Lara Atkinson, a researcher with the South African Environmental Observation Network (SAEON), leading the invertebrate monitoring component. The individual male specimen was sent for identification to the junior author who concluded that the specimen might represent an undescribed species of *Paragiopagurus* Lemaitre, 1996, but without additional specimens he was unable to make a final determination. Subsequently, during the 2015 and 2016 DAFF West Coast demersal surveys, numerous additional specimens were collected on request of the senior author, and proved to be conspecific with the first male specimen obtained by Dr. Atkinson. A detailed taxonomic study of all these specimens showed that indeed, they represent a new species of *Paragiopagurus* that co-occurs with the two common parapagurid species in the DAFF demersal research surveys, although in a comparatively confined area on the West Coast. Herein we fully describe and illustrate this new species, including colour photographs. Furthermore, to improve understanding of the parapagurid fauna occurring on the South African continental shelf, we compare this new species with the other two co-existing parapagurids, *S. dimorphus* and *P. bouvieri*. For the first time, live colour information is provided for the latter two hermit crab species. In combination, this diagnostic information on the three most common South African deep-water hermit crabs will facilitate improved accuracy in identification of the species, as well as future monitoring and ecological studies.

The systematics and taxonomy of deep-water hermit crabs of the family Parapaguridae has been revised in a number of broad studies over the last three decades. The family currently includes 91 species classified in 10 genera, of which five are monotypic (Lemaitre 1989; 1993; 1996; 1998; 1999; 2004a; 2004b; 2013; 2014; Osawa 1995; McLaughlin et al. 2010). The new species described herein within *Paragiopagurus* Lemaitre, 1996, is the 25^th^ known for this genus. The other two genera represented in the bycatch of demersal research surveys, *Parapagurus* Smith, 1879, and *Sympagurus* Smith, 1883, each contain 17 species. Although many species of parapagurids are known to occur in the western Indian Ocean and vicinity of the east African coast, only eight species in four genera have previously been documented specifically from South Africa: *Oncopagurus africanus* (de Saint Laurent, 1972), *Parapagurus andreui* Macpherson, 1984, *P. bouvieri*, *P. richeri* Lemaitre, 1999, *P. stenorhinus* Lemaitre, 1999, *Strobopagurus sibogae* (de Saint Laurent, 1972), *Sympagurus dimorphus*, and *S. trispinosus* (Balss, 1911). In a recent catalogue of decapods, Emmerson (2016a, b) did list 13 species of parapagurids from the broad region that encompasses Namibia, South Africa and Mozambique, including two species of *Paragiopagurus*; however, the two latter species have only been reported from the Valdivia Bank, off Namibia. Thus, the new species of *Paragiopagurus* described herein represents the first report of a species of *Paragiopagurus* in waters of South Africa.

Several earlier reports of parapagurids from South Africa have been corrected in various taxonomic revisions of species in this family, as follows. Lemaitre (1989, 1999) concluded that reports by Kensley (1969, 1974, 1977) of *Parapagurus pilosimanus* Smith, 1879 actually represent *P. bouvieri*. The subspecies *Parapagurus pilosimanus bouvieri* proposed by de Saint Laurent (1972) in her division of the genus *Parapagurus*, and listed by Kensley (1981) in his zoogeographic study of Southern African decapods, was elevated to species status by Lemaitre (1989, 1999). *Parapagurus kilburni* Kensley, 1973, described from off Durban, South Africa, and subsequently listed by Kensley (1981), was determined by Lemaitre (2004a) to be conspecific, and thus a junior synonym, of *Strobopagurus sibogae*.

## Materials and methods

Since 2011, targeted invertebrate specimens retained in the research trawl nets were collected during the DAFF demersal research abundance surveys, using a German otter trawl design with various configurations, and a 75 mm mesh cod-end fitted with a 35 mm mesh liner. Trawls were deployed for 30 minutes (bottom time) over all feasible habitats on the South African shelf (for detailed methods see Atkinson et al. 2011). During the 2015 research surveys, hermit crabs were pre-sorted on board by scientific staff, and all specimens of *S. dimorphus* and *P. bouvieri* were separated. Three male specimens with “green eyes” were obtained during the 2015 surveys. During the 2016 research surveys, a subsample of approximately 100 hermit crab specimens from each trawl were separated and frozen for further identification at the University of Cape Town (UCT). Three additional males and 23 females with “green eyes” were obtained from trawls at two West Coast stations during the 2016 surveys. All specimens with “green eyes” were found to be the new species of *Paragiopagurus* herein described. No specimens with “green eyes” were found in trawls from the South Coast. Live images of *S. dimorphus* and *P. bouvieri* were taken in the laboratory at the University of Cape Town, and in a photographic tank on board of the RS Africana during an additional South Coast spring survey in September/October of 2016.

The µCT scan of the holotype of the new species of *Paragiopagurus* was performed at the CT Scanner Facility at Stellenbosch University, South Africa, using a General Electric Phoenix V|Tome|X L240 with NF180 option (du Plessis et al. 2016). The specimen was defrosted and placed on top of a plastic rod with dense polystyrene foam as a platform, and consecutively scanned at an X-ray voltage of 100 kV and 100 µA, and a resolution of 35 µm. Images were recorded in 3200 steps in one full rotation of the sample averaging two image acquisitions at every step. Using a detector shift function between images reduced ring artifacts. The projection images were reconstructed using the system-supplied General Electric Datos reconstruction software, which were subsequently utilized for the visualization of the right cheliped using Volume Graphics VGStudioMax 3.1. (Heidelberg, Germany).

Illustrations were drawn using a Wild stereomicroscope equipped with a camera lucida, and digitally traced in Inkscape 0.91 (www.inkscape.com). Colour photographs were processed in Gimp 2.8 (www.gimp.com).

Specimens examined in this report are deposited in the Iziko South African Museum, Cape Town, South Africa (SAMC), the National Museum of Natural History, Smithsonian Institution, Washington DC (USNM), as well as in the Lee Kong Chian Natural History Museum, Singapore (ZRC). Morphological terminology for parapagurids is that used by Lemaitre (2013). Measurements of specimens, in millimeters (mm), listed in the material examined sections are for shield length (SL), taken from the tip of the rostrum to the midpoint of the posterior margin of the shield. Other abbreviations used are: ovig: ovigerous; SCDSA: South Coast Demersal Survey Autumn; SCDSS: South Coast Demersal Survey Spring; WCDSS: West Coast Demersal Survey Summer; sta: station; and in the material examined sections, months are abbreviated by the first three letters.

Muscular tissue, usually from the merus of the right cheliped, was extracted from freshly frozen specimens, placed in 96% ethanol, and sent to the South African Institute for Aquatic Biodiversity (SAIAB). At SAIAB, DNA extractions were carried out using a standard “salting out – ethanol precipitation” protocol (Sunnucks and Hales 1996), followed by the amplification of the ‘barcoding’ (Hebert et al. 2003) fragment of the cytochrome *c* oxidase subunit I (CO1) gene for each sample by Polymerase Chain Reaction (PCR), using the universal invertebrate primers (LCOI-1490 and HCOI-2198) of Folmer et al. (1994), or their degenerate variants (dgLCO1490 and dgHCO2198; Meyer 2003). PCR recipes and conditions followed Meyer (2003) and Gouws et al. (2015), with annealing performed at 48 °C for the latter. Successful amplification was determined by visualising products under UV light, following electrophoresis in 1% agarose gels, stained with ethidium bromide, in a TBE buffer. PCR products were purified with an Exonuclease I – Shrimp Alkaline Phosphate (Exo/SAP, ThermoFisher Scientific) protocol (Werle et al. 1994), sequenced in both the forward and reverse directions using BigDye v3.1 (Applied Biosystems, Austin, Texas) terminator chemistry and analyzed on an ABI-Hitachi 3500 Genetic Analyser (Applied Biosystems) at SAIAB. The resulting sequences were checked against their chromatograms for misreads and sequencing errors using ChromasLITE (Technylesium). Sequences were aligned, edited and the consensus DNA barcode compiled using Lasergene SeqMan Pro 9 (DNASTAR, Madison, Wisconsin). Barcodes were uploaded to the SeaKeys (SEAKY) project on BOLD (www.boldsystems.org; Ratnasingham and Hebert 2007) and were submitted to GenBank. For a number of specimens, tissues were submitted to the Canadian Centre for DNA Barcoding, Biodiversity Institute of Ontario, University of Guelph, for barcoding. These data were also uploaded to SEAKY on BOLD. For future reference and studies, the database gene codes are included under each species.

## Results

### Systematic account

#### Family Parapaguridae Smith, 1882

##### Genus *Paragiopagurus* Lemaitre, 1996

###### Paragiopagurus atkinsonae sp. n.

Taxon classificationAnimaliaDecapodaParapaguridae

http://zoobank.org/833540CC-B266-4010-A401-E7CA010CDE6A

**Figure 2.**
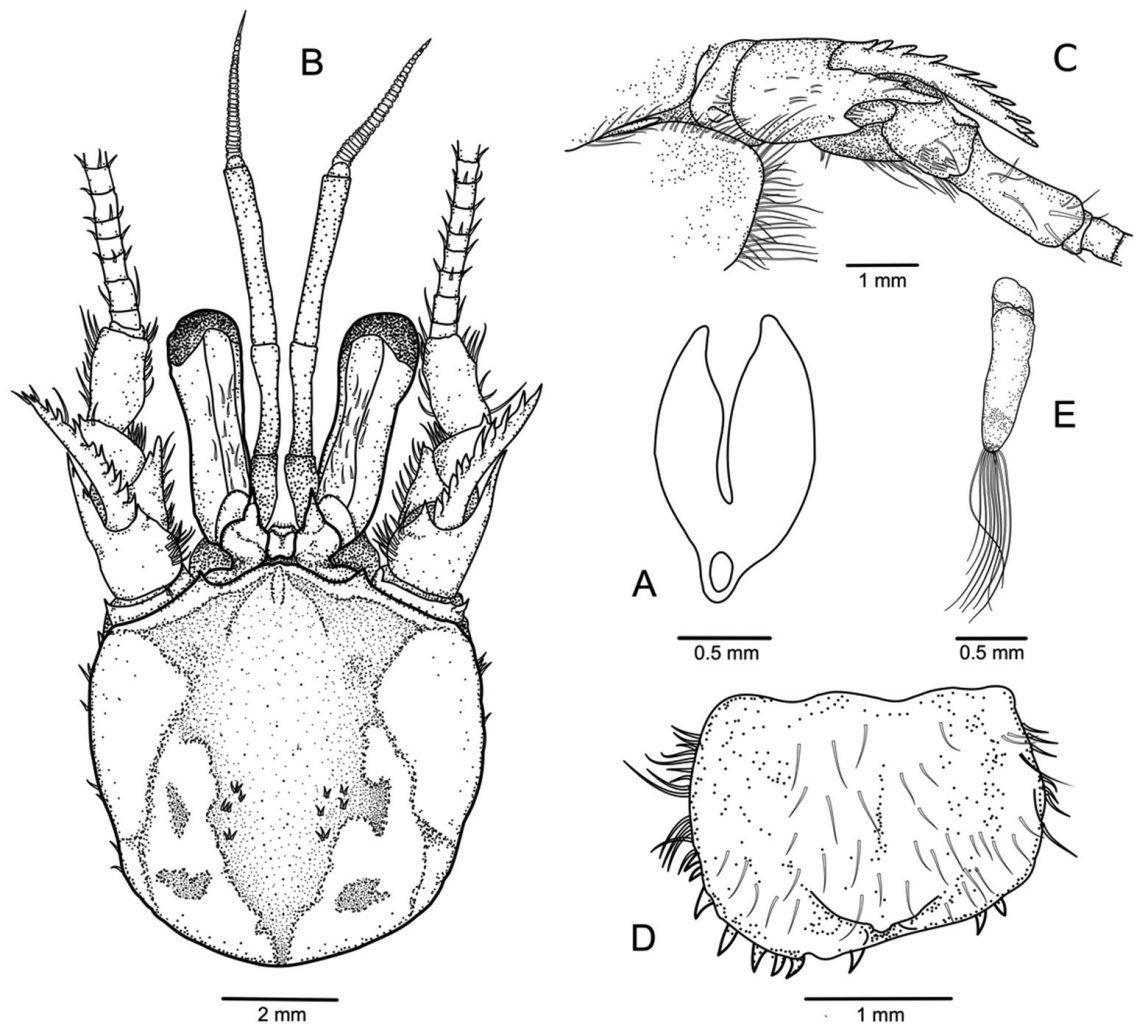
*Paragiopagurus atkinsonae* sp. n., South Africa, West Coast: **A** male paratype 7.0 mm, WCDSS2015 (USNM 1292084); **B–D** male holotype 7.0 mm, WCDSS2016 (USNM 1292083). **A** gill lamella of posterior-most arthrobranch **B** shield and cephalic appendages, dorsal view **C** right antennal peduncle and branchiostegite, lateral view **D** telson, dorsal view **E** left pleopod 2, lateral view.

**Figure 3.**
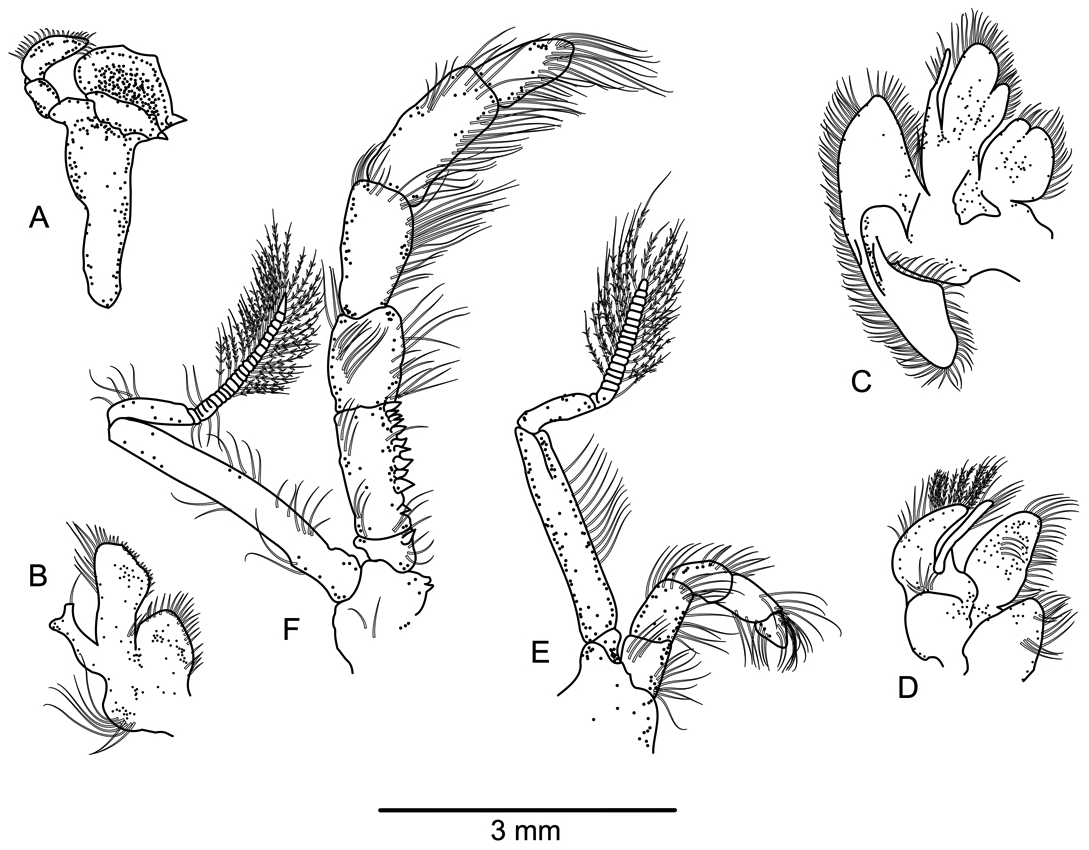
*Paragiopagurus atkinsonae* sp. n., South Africa, West Coast, male paratype 7.0 mm, WCDSS2015 (USNM 1292084). Left mouthparts, internal view. **A** mandible **B** maxillule **C** maxilla **D** first maxilliped **E** second maxilliped **F** third maxilliped.

**Figure 4.**
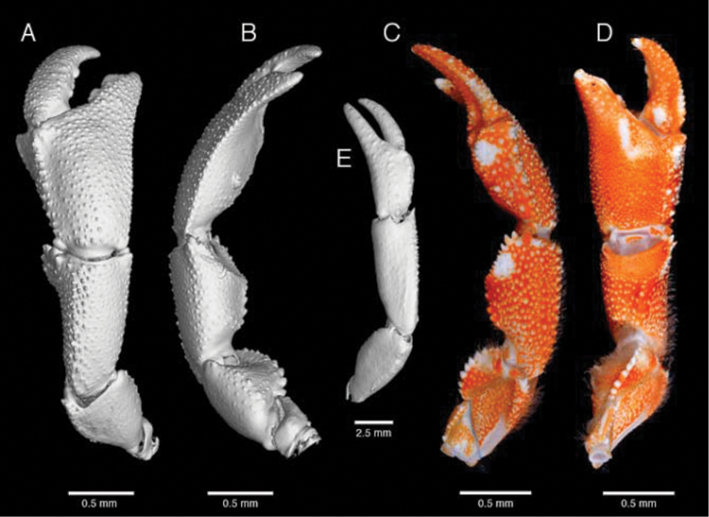
*Paragiopagurus atkinsonae* sp. n., South Africa, West Coast: **A, B, E** microCT scans, male holotype 7.0 mm, WCDSS2016 (USNM 1292083); **C, D** photographs, male paratype 6.8 mm, WCDSS2016 (SAMC MB-A066815). Right cheliped: **A** dorsal view **B** lateral view **C** mesial view **D** ventral view. Left cheliped: **E** dorsal view.

**Figure 5.**
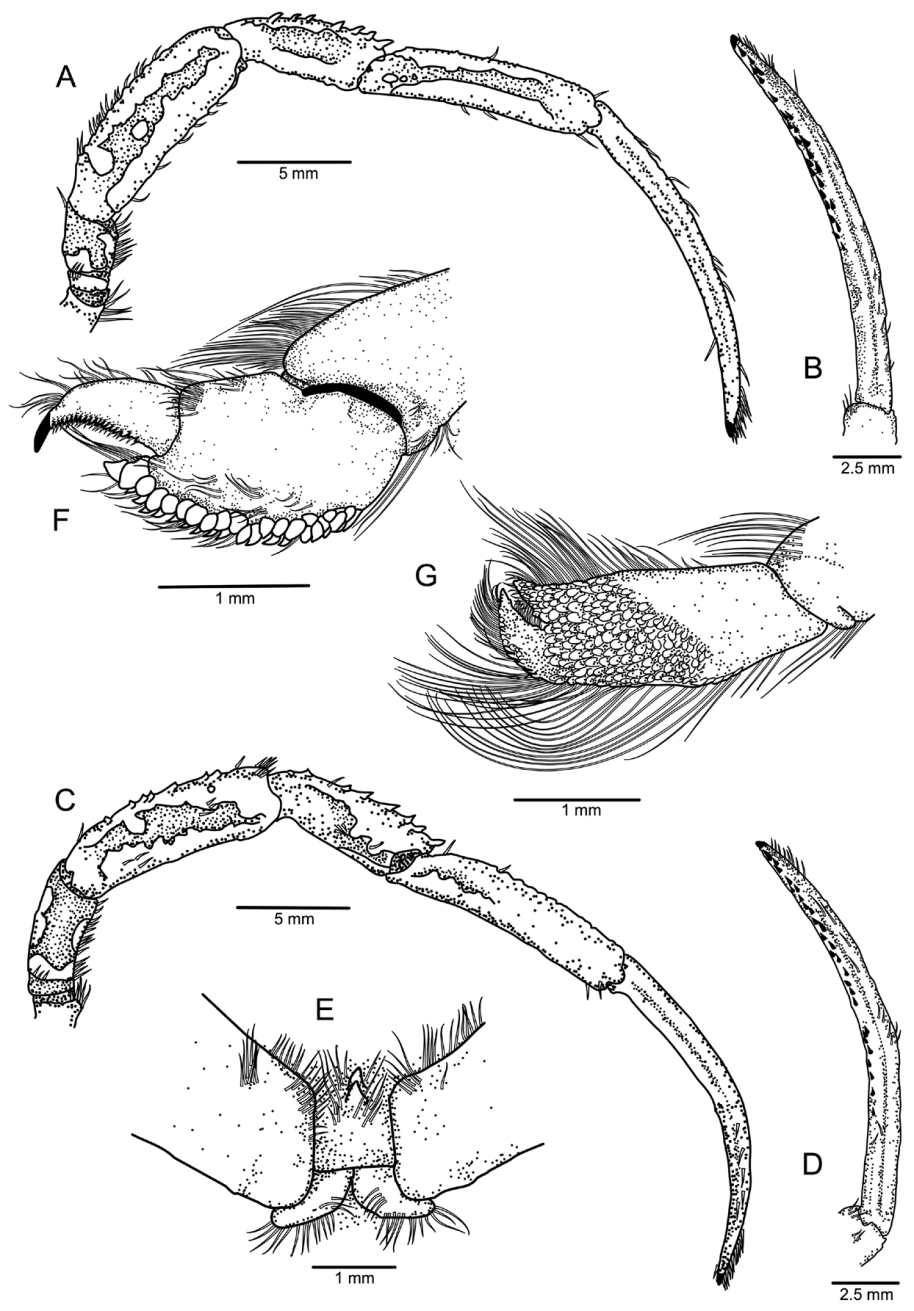
*Paragiopagurus atkinsonae* sp. n., South Africa, West Coast, male holotype 7.0 mm, WCDSS2016 (USNM 1292083). **A** pereopod 2, lateral view **B** dactyl of same, mesial view **C** pereopod 3, lateral view **D** dactyl of same mesial view **E** sternite XII and basal portion of coxae of pereopods 3, ventral view **F** propodus and dactyl of pereopod 4, lateral view **G** propodus and dactyl of pereopod 5, lateral view.

**Figure 6.**
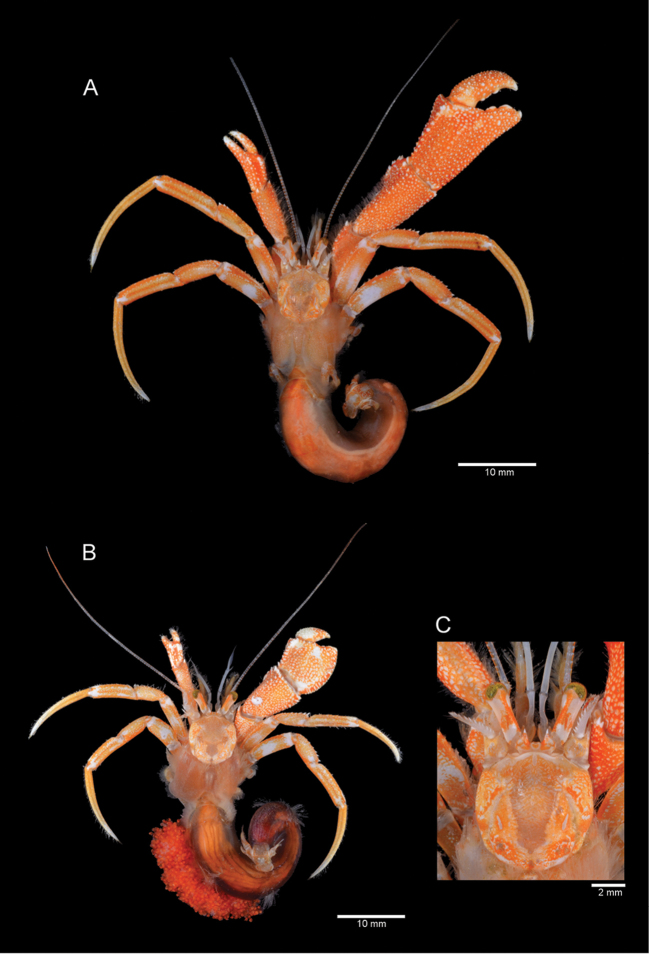
Colouration (in life or fresh). *Paragiopagurus atkinsonae* sp. n., South Africa, West Coast: **A** male holotype 7.0 mm, WCDSS2016 (USNM 1292083) **B** ovig. female 6.4 mm, WCDSS2016 (SAMC MB-A066816) **C** ovig. female 6.8 mm, WCDSS2016 (SAMC MB-A066809), shield and cephalic appendages, dorsal view.

Figs 2 , 3 , 4 , 5 , 6

####### Type material.

Holotype: male 7.0 mm, South Africa, West Coast, WCDSS2016, CCH008, sta D00723–3243, S31°52.81', E16°57.12', 265 m, 11 Mar 2016 (USNM 1292083).

Paratypes: *South Africa, West Coast*. WCDSS2012, AFR279: 1 male 7.6 mm [with zoanthid symbionts], sta A32208–3233, S31°39.79', E17°02.79', 259 m, 24 Jan 2012, coll. L. Atkinson (USNM 1292086). WCDSS2015, AND004: 1 male 7.6 mm, sta C0416–3258, S32°08.05', E17°08.52', 230 m, 26 Feb 2015 (USNM 1292080); 1 male 7.0 mm (USNM 1292084), 1 male 6.2 mm (SAMC MB-A066814), sta C430–3237, S31°42.07', E16°58.53', 277 m, 1 Mar 2015. WCDSS2016, CCH008: 1 male 6.8 mm, sta D00724, S32°03.18', E17°03.11', 243 m, 11 Mar 2016 (SAMC MB-A066815); 1 male 7.8 mm (USNM 1292082), 3 females 6.4–7.0 mm (USNM 1292081), 4 females 5.9–7.1 (USNM 1292085), 1 ovig. female 6.8 mm (SAMC MB-A066809), 1 ovig. female 5.9 mm (SAMC MB-A066810), 1 ovig. female (SAMC MB-A066811), 3 ovig. females 6.6–7.2 mm, 7 females 7.2–8.0 mm (SAMC MB-A066812), 2 ovig. females 6.7–7.3 mm (SAMC MB-A066813), 1 ovig. female 6.4 mm (SAMC MB-A066816), sta D00726–2446, S32°22.98', E17°27.78', 199 m, 11 Mar 2016.

####### Description.

Eleven pairs of biserial (Fig. 2A), or at most weakly divided quadriserial gills. Shield (Fig. 2B, 6C) about as broad as long; dorsal surface nearly naked or with scattered short setae, with weakly- to moderately-calcified median region extending from anterior margins of rostrum, anterior and lateral projections, to about proximal 0.2 length of shield; anterior, lateral and posterior margins with short setae. Rostrum broadly rounded, with short mid-dorsal ridge. Anterior margins weakly concave. Lateral projections subtriangular, armed with short terminal spine. Anterolateral margins sloping. Ventrolateral margin usually with small spine. Posterior margin broadly rounded. Anterodistal margin of branchiostegite rounded, unarmed, setose.

Ocular peduncles (Fig. 2B) about half, or slightly more than half, length of shield, each with longitudinal row of short setae on dorsal surface. Corneas weakly dilated. Ocular acicles subtriangular, about 0.3 as long as ocular peduncles, each terminating in strong, simple spine; separated basally by about 0.6 the width of 1 acicle.

Antennular peduncles exceeding distal margin of corneas by 0.8–0.9 length of ultimate segment; ventral flagellum with 5–7 articles. Ultimate segment twice, or more than twice, as long as penultimate, with scattered setae dorsally. Basal segment with strong ventromesial spine; lateral face with distal subrectangular lobe armed with 1 or 2 spines, and strong spine proximally.

Antennal peduncles (Fig. 2C) reaching to about distal margin of corneas. Fifth segment unarmed, with longitudinal row of setae on lateral and mesial margins. Fourth segment with strong spine on dorsolateral distal angle. Third segment with strong ventromesial distal spine. Second segment with dorsolateral distal angle produced, terminating in strong, simple spine extending to about half length of acicle and having 2 or 3 small spines dorsally; mesial margin with spine on dorsodistal angle. First segment with lateral surface armed with small spine; ventromesial angle not strongly produced, armed with 1–3 small, blunt spines. Antennal acicle slightly curved outward (dorsal view), overreaching proximal margin of cornea, but not exceeding distal margin of cornea, terminating in strong spine; mesial margin with row of about 10 strong spines of similar size and set at about 45^0^ to longitudinal axis of acicle. Flagellum exceeding distal margin of extended right cheliped, nearly naked, or with scattered, short setae less than 1 flagellar article in length.

Mandible (Fig. 3A) with 3-segmented palp. Maxillule (Fig. 3B) with external lobe of endopod moderately-well developed, internal lobe with 1 long setae. Maxilla (Fig. 3C) with endopod well exceeding distal margin of scaphognathite. First maxilliped (Fig. 3D) with endopod exceeding distal margin of exopod. Second maxilliped (Fig. 3E) without distinguishing characters. Third maxilliped (Fig. 3F) with crista dentate with about 10 corneous teeth, decreasing in size distally; basis with 1 dorsomesial corneous tooth; coxa with 1 or 2 mesial teeth.

Chelipeds markedly dissimilar, proportions strongly affected by size and sexual dimorphism, males growing distinctly longer right chelipeds with narrower chela, than females. Right cheliped (Figs 4, 6A, B) massive; in males, about 1.5 times as long as left cheliped and 4.3 times as long as SL; in females, about 1.3 times as long as left cheliped and 3 times as SL; dorsal surfaces covered with sparse or inconspicuous short, simple or plumose setae. Chela operculate, somewhat dorsoventrally flattened, less so in males; males about twice as long as wide, or in females about 1.3 times as long as wide. Fingers moderately curving mesioventrally, each terminating in small corneous claw, dorsal surfaces covered with numerous small, blunt to sharp tubercles or spines, ventral surfaces covered with small tubercles; cutting edge of dactyl with 2 larger calcareous teeth and several small teeth in between, distal row of small fused corneous teeth; cutting edge of fixed finger with 2 large, rounded calcareous teeth and several small calcareous teeth distally and proximally. Dactyl longer (female), or shorter (male), than mesial margin of palm, set at oblique angle to longitudinal axis of palm; mesial margin well defined by longitudinal row of spines or tubercles; proximal half of ventromesial face strongly concave. Fixed finger basally much broader in females than in males. Palm distinctly broader than long in females, or usually distinctly longer than broad in males; dorsal surface covered with numerous small tubercles or spines; lateral margin well defined by row of small tubercles or spines; dorsomesial margin with row of irregular spines (less strong in males); mesial face strongly sloping, slightly concave (less so in males), covered with small tubercles; ventromesial margin weakly delimited (less so in males) by row of low tubercles or spines; ventral surface nearly flat or weakly convex, with small tubercles or spines less numerous than on dorsal surface. Carpus similar to chela in general armature and setation, subtriangular in cross-section, longer in males than in females; dorsal surface covered with numerous small tubercles or spines, generally spines sharper in females than in males; dorsal margin with irregular row of spines, dorsodistal margin armed with strong (females) or weak (males) spines, increasing in size mesially; ventrolateral margin well defined (more so in females) by row of spines increasing in size distally; ventromesial distal margin somewhat expanded, wing-like, armed with row of strong spines. Merus subtriangular in cross-section, dorsal margin unarmed, or with low tubercles and row of short setae, and strong dorsodistal spine; lateral surface with minute tubercles; ventrolateral margin with row of blunt spines distally; mesial surface flat, unarmed, ventromesial margin with row of strong, mostly blunt spines; ventral surface smooth or with very low tubercles. Ischium with ventrolateral row of small spines, and moderately long setae mesioventrally. Coxa with row of small spines on ventrolateral distal margin and ventrodistal row of long setae.

Left cheliped (Figs 4E, 6A, B) generally well calcified, reaching to base of dactyl (females), or mid-length of palm (males), of right cheliped. Fingers weakly bent lateroventrally, gaping slightly when closed, each terminating in sharp corneous claw; dorsal and ventral surfaces unarmed, except for few tufts of short setae; cutting edges each with closely-set small, corneous teeth. Dactyl slightly longer than palm; proximal half of ventromesial face slightly concave. Palm longer than wide; dorsal surface with 2 median rows of small, low tubercles, and sparse tufts of short setae, somewhat depressed medially; dorsomesial margin with row of small tubercles or spines; dorsolateral margin rounded; ventral surface unarmed except for scattered setae. Carpus with moderately dense setation on dorsal, lateral and mesial surfaces; dorsal margin with irregular row of small tubercles or spines, and strong dorsodistal spine; lateral and mesial faces unarmed except for setae, and strong spine on lateroventral distal angle; ventral surface smooth, at most with tufts of sparse setae. Merus unarmed except for minute tubercles on lateral, mesial and ventral faces, and dense setation on dorsal ventromesial margins. Ischium unarmed and smooth except for dense setae on ventral surface. Coxa at most with minute spines on ventromesial distal margin and row of setae on ventrodistal margin.

Ambulatory legs or pereopods 2 and 3 (Figs 5A–D, 6A, B) similar from right to left, except for slightly longer meri on right; usually exceeding right cheliped by about 0.2 length of dactyl of legs when fully extended. Dactyl about 1.5–1.9 as long as propodus, broadly curved, terminating in sharp corneous claw; dorsal margin mostly with short setae, except for distal row of bristle-like setae; ventromesial margin (Fig. 5B, D) armed with 2 or 3 irregular rows of short, corneous spinules and usually terminating as single row near claw; lateral and mesial face with shallow, longitudinal sulcus on proximal half, deeper on mesial face. Propodus nearly naked; dorsal margin with setae usually arising from low tubercles. Carpus nearly naked, or with sparse short setae; dorsal margin armed with row of distinct, well-spaced small spines (stronger on pereopod 2) increasing slightly in size distally, and small dorsodistal spine. Merus unarmed except for scattered setae on dorsal margin. Ischium with dorsal and ventral row of setae. Coxa unarmed except for 1 or 2 minute spines on ventromesial proximal angle (pereopod 2 only) and ventromesial row of setae. Anterior lobe of sternite XII (of pereopods 3; Fig. 5E) subtriangular, setose, and terminating in simple or more frequently bifid spine.

Pereopod 4 (Fig. 5D) subchelate. Dactyl broadly curved, terminating in sharp, corneous claw, with ventrolateral row of small corneous teeth increasing in size distally. Propodus longer than wide; rasp consisting of 2 or 3 rows of rounded scales. Carpus with long setae on dorsal margin. Merus with rows of long setae on dorsal, ventromesial and ventrolateral margins.

Pereopod 5 (Fig. 5F) chelate. Propodal rasp extending slightly beyond mid-length of segment. Dactyl with row of minute, rounded scales on ventrolateral surface.

Uropods and telson asymmetrical. Telson (Fig. 2D) lacking transverse sutures separating anterior and posterior lobes; dorsal surface with scattered short setae; lateral margins with moderately long (left) and short (right) setae; posterior lobes separated by narrow, median cleft, terminal margins rounded, armed with row of 15–8 (left lobe) or 10–12 (right lobe) short corneous spines, some slightly curved.

Males lacking first gonopods; with unpaired left pleopods 2–5, of which pleopod 2 (Fig. 2E) is 2-segmented, uniramous, and other pleopods biramous. Females with unpaired pleopods 2–5, with well-developed rami on pleopods 2–4, and short endopod on pleopod 5.

**Colour (in life; Figs 4C, D, 6A–C).** Shield and cephalic appendages mottled orange and cream to white. Ocular peduncles white with basally and distally broadened dorsomedian orange stripe; orange pattern extending to ventromesial face just below midlength of ocular peduncle. Corneas usually green. Ocular acicles mottled orange with white spines. Chelipeds orange-red, with white tubercles and spines. Right chela often with dactyl and fixed finger each with cream patch proximally at about midline, fingertips white; propodus, merus and carpus with distinct cream to white spot on dorsomesial distal angle. Left chela with cream patches of different size, fingertips white. Ambulatory legs orange overall; dactyl light orange, distally cream; propodus with cream patch on distolateral and distomesial angles, lateral face with dark orange stripe; carpus orange overall, with lighter orange medially on lateral face. Merus with white band distally, large white patch on proximal half of lateral face, and darker orange on dorsodistal margin. Uropods and telson mottled orange and cream. Pleon orange, in some females dark red ventrally due to gonads with unspawned eggs. Eggs bright red.

####### Habitat.

Occupying shells created by colonies of *Epizoanthus* sp. that incorporate sand grains in their tissue and form a carcinoecia that completely covers a minute gastropod shell. This *Epizoanthus* sp. appears the same to that frequently used by *Sympagurus dimorphus* in the South African region.

####### Distribution

**(Fig. 8).** Known so far only from a small portion of the west coast of South Africa, between 31°42'S and 32°23'S, in a depth range of 199–277 m.

####### Etymology.

This species is named after Dr. Lara Atkinson, a researcher from the South African Environmental Observation Network (SAEON), Egagasini Node for marine-offshore systems, who first noticed the presence of this new species and collected the first specimen. The name honours her research efforts to understand the benthic marine fauna of South Africa, and acknowledges the major role she played in organizing sampling of additional material of this new species.

####### Common name.

“Green-eyed hermit crab”.

####### Genetic data.

Sta D00723-3243, S31°52.81', E16°57.12', 265 m, male 7.0 mm (holotype), BOLD: SEAKY1181-17 (USNM 1292083). Sta D00726–2446, S32°22.98', E17°27.78', 199 m, ovig. female 6.8 mm, BOLD: SEAKY1181-17 (MB-A066809); ovig. female 5.9 mm, BOLD: SEAKY1183-17 (MB-A066810); male 6.8 mm, BOLD: SEAKY1180-17 (MB-A066815).

####### Variations.

In males with SL > 7.0 mm, the right cheliped (merus to dactyl) ranges from 3.6–4.8 times as long as the shield, and the chela varies from 1.7–2.4 as long as wide. In females with SL > 5.9 mm, the right cheliped (merus to dactyl) ranges from 2.6–3.2 times as long as the shield, and the chela varies from 1.3–1.6 as long as wide. The spination of both right and left chelae tends to be sharper, and stronger in females.

####### Remarks.

Three characters present in *Paragiopagurus atkinsonae* sp. n. exemplify the morphological evolutionary tendencies that in general are observed (Lemaitre 2013) in species of *Paragiopagurus*. These three characters are: biserial gills that are, at most, weakly divided distally; the drastic sexual dimorphism exhibited on the right cheliped; and in males, the complete loss of paired first and second pleopods modified as gonopods. In sharing these three characters, this new species is most similar to *P. ventilatus* Lemaitre, 2004c, a northwestern Pacific species that is known to associate with hydrothermal vents in the northeastern coast of Taiwan and the Mariana Trough (Lemaitre 2004c; Komai et al. 2010). Additionally, both species share a rare armature condition in parapagurids for the ventromesial margin of the dactyls of the ambulatory legs, being armed in this new species with two or three irregular rows of numerous corneous spinules instead of a single regular row of relatively few spines, as in other species of this genus. In other respects, however, these two species are markedly different. In *P. atkinsonae* sp. n. the fourth antennal segment is armed with a spine on the dorsolateral distal angle, whereas in *P. ventilatus* the fourth segment is unarmed; the antennal flagella is nearly naked or with scattered short setae, whereas in *P. ventilatus* the flagella are densely covered with long setae; the third maxillipeds and basal segments of the chelipeds lack dense plumose setae, whereas in *P. ventilatus* these are present; the propodal rasp of pereopod 4 has two or three rows of ovate scales, whereas in *P. ventilatus* the rasp has only one row of ovate scales; the telson is weakly asymmetrical, whereas in *P. ventilatus* the telson is strongly asymmetrical. Furthermore, *P. atkinsonae* sp. n. is not associated with hydrothermal vent habitats, whereas *P. ventilatus* has been found exclusively in or close to vent habitats (Lemaitre 2004c; Komai et al. 2010).

In addition to *Paragiopagurus atkinsonae* sp. n., there are seven other species of *Paragiopagurus* in which the male lacks paired first and second gonopods: *P. trilineatus* Lemaitre, 2013, *P. bicarinatus* (de Saint Laurent, 1972), *P. hirsutus* (de Saint Laurent, 1972), *P. acutus* (de Saint Laurent, 1972), *P. ruticheles* (A. Milne-Edwards, 1891), *P. hobbiti* (Macpherson, 1983), and *P. ventilatus*. The complete pleopod condition in the male for all these species is the same, i.e., presence of left unpaired pleopods 2–5. Pleopod 2 is uniramous, 2-segmented, with a short distal segment, and pleopods 3–5 are biramous. In both sexes of *P. atkinsonae* sp. n., the propodal rasp of pereopod 4 has two or three rows of ovate scales, a condition similar to that of three other congenerics: *P. trilineatus*, *P. pilimanus* (A. Milne-Edwards, 1880), and *P. tuberculosus* (de Saint Laurent, 1972). Other than the development of pleopods in the male, and the number of rows of scales on the propodal rasp of the pereopod 4, *P. atkinsonae* sp. n. differs significantly from all those species (see Lemaitre 2013).

When using Lemaitre’s (2013) species identification key for specimens of *Paragiopagurus atkinsonae* sp. n., the user will reach couplet 19. To accommodate this new species to that key, couplet 19 can be replaced with the following two new couplets 19 and 20 (and changing the numbers of Lemaitre’s couplets 20–23 by +1):

**Table:** 

19	Ventromesial margins of ambulatory legs (pereopods 2, 3) armed with several irregular rows of numerous corneous spinules	**20**
–	Ventromesial margins of ambulatory legs (pereopods 2, 3) armed with single, regular row of corneous spinules	**21**
20	Propodal rasp of pereopod 4 with 2 or 3 rows of ovate scales; antennal flagella naked or with scattered short simple setae; fourth antennal segment armed with spine on dorsolateral distal angle; telson weakly asymmetrical	***Paragiopagurus atkinsonae* sp. n.**
–	Propodal rasp of pereopod 4 with 1 row (at least distally) of ovate scales; antennal flagella densely covered with long mostly plumose setae; fourth antennal segment lacking spine on dorsolateral distal angle; telson strongly asymmetrical	***P. ventilatus***

##### Genus *Sympagurus* Smith, 1883

###### Sympagurus dimorphus

Taxon classificationAnimaliaDecapodaParapaguridae

(Studer, 1883)

Fig. 7A, B , 8

Primary synonyms: Eupagurus dimorphus Studer, 1883: 24, figs 11, 12 (type locality: South Atlantic Ocean, South Africa, off Cape of Good Hope, S.M.S. “Gazelle”, 34°13.6'S, 15°00.7'E, 211 m).
Parapagurus brevimanus Balss, 1911: 4, fig. 5.
? Eupagurus modicellus Stebbing, 1914: 255, pl. 26, fig. D. (See “General distribution”).
Sympagurus var. *arcuatus johnstoni* Hale, 1941: 279, fig. 13a–d.
Sympagurus var. *arcuatus mawsoni* Hale, 1941: 280, fig. 14a–c.

####### Material examined.

*South Africa, West Coast*. WCDSS2012, AFR279: 4 males 9.5–12.0 mm, 1 ovig. female 8.1 mm, sta A32144–4116, S32°18.26', E16°18.53', 369 m, 11 Jan 2012 (SAMC MB-A066808). WCDSS2015, AND004: 1 ovig. female 9.7 mm [inside stomach of Monk fish], sta C0400–3330, S33°55.08', E17°39.26', 285 m, 20 Jan 2015 (SAMC MB-A066807); 4 males 10.0–12.0 mm, 5 ovig. females 7.7–9.1 mm, 1 female 9.4 mm, sta C0367–3336, S33°58.11', E17°52.51', 220 m, 9 Feb 2015 (SAMC MB-A066801); 1 male 7.4 mm, sta C0379-3130, S36°34.74', E20°38.10', 12 Feb 2015 (SAMC MB-A066805); 1 female 7.1 mm, sta C0458-5008, S29°57.54', E14°49.40', 448 m, 8 Mar 2015 (SAMC MB-A066803). WCDSS2016, CCH008: 1 male 7.8 mm, sta D00640, S31°28.02', E16°05.64', 470 m, 21 Feb 2016 (SAMC MB-A066806); 1 male 11.2 mm, sta D00726–2446, S32°22.98', E17°27.78', 199 m, 11 Mar 2016 (SAMC MB-A066492).

*South Africa, South Coast*. SCDSA 2015, AND005: 1 male 14.6 mm, sta D0520-4071, S36°27.78', E21°53.58', 401 m, 20 Apr 2015 (SAMC MB-A066839); 1 male 12.0 mm (SAMC MB-A066840), 1 male 13.2 mm (SAMC MB-A066841), sta D00521–4043, S36°25.50', E21°27.12', 192 m, 20 Apr 2015; 1 male 4.9 mm, sta D00540-6542, S35°21.30', E22°49.98', 585 m, 26 Apr 2015 (SAMC MB-A066833); 1 male 12.7 mm, sta D00561-6671, S34°05.22', E26°55.68', 466 m, 1 May 2015, (SAMC MB-A066818); 1 male 10.3 mm, sta D00565-4224, S34°10.20', E26°46.38', 425 m, 2 May 2015 (SAMC MB-A066823); 1 male 6.5 mm, 1 female 7.5 mm, sta D00582–4153, S34°54.96', E23°22.08', 210 m, 7 May 2015 (SAMC MB-A066820). SCDSA 2016, CCH009: 1 male 11.3 mm, sta D00757-4020, S36°49.19', E20°33.72', 538 m, 4 May 2016 (SAMC MB-A066802); 2 males 10.1–10.4 mm, 3 ovig. females 6.8–7.6 mm, sta D00812–4174, S34°46.80', E24°12.30', 196 m, 19 May 2015 (SAMC MB-A066804); 1 male 8.8 mm (SAMC MB-A066490), 1 ovig. female 9.4 mm (SAMC MB-A066491), sta D00819, S34°52.32', E23°35.70', 195 m, 21 May 2016.

####### Diagnosis, taxonomy, larval and juvenile morphology.

See Lemaitre (1989; 1990; 1996; 2000; 2004b), Lemaitre and McLaughlin (1992), and Poore (2004).

**Figure 7.**
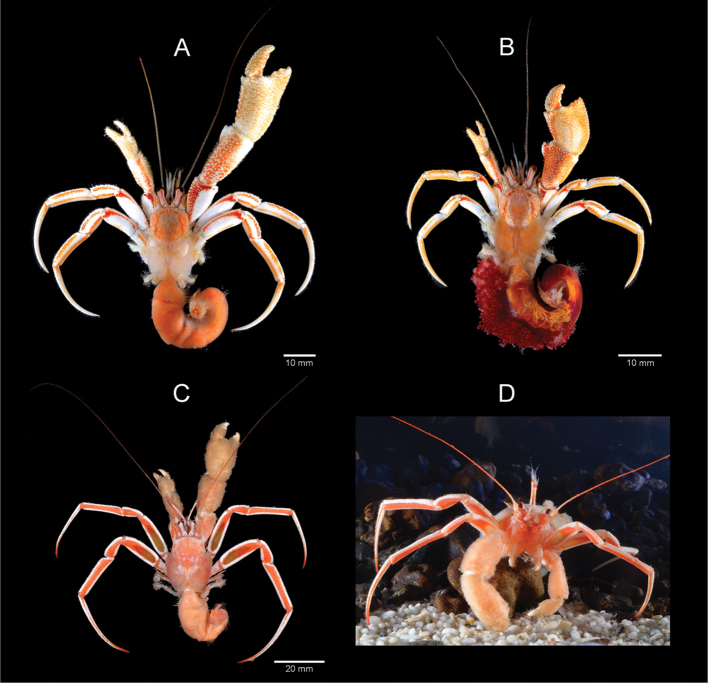
**A, B** *Sympagurus dimorphus* (Studer, 1883), South Coast **C, D** *Parapagurus bouvieri* Stebbing, 1910, West Coast (**C**), South Coast (**D**). **A** male 11.2 mm SCDSA 2016 (SAMC MB-A066492) **B** ovig. female 9.4 mm SCDSA 2016 (SAMC MB-A066491) **C** male 12.2 mm, WCDSS (SAMC MB-A066432) **D** male 10.6 mm, SCDSS2016 (SAMC MB-A066794), front view of live specimen in aquarium, using zoanthid (*Epizoanthus* sp.) carcinoecia.

**Colour (in life; Fig. 7A, B).** Until now, information on colour of *Sympagurus dimorphus* had been based on three published photographs taken of live specimens inside their gastropod housing (Lemaitre 2000, pl. 7; 2004b, fig. 35a; Poore 2004: pl. 17c), and formalin-preserved specimens with patterns still visible (Lemaitre 2004b). Although the basic colour pattern can be discerned in those photographs, the specimens used have only partially visible body parts, and furthermore, the exposures of the images show somewhat distorted colour tones. Herein, we present for the first time a high quality colour photograph of the entire body removed from it’s housing of a freshly caught male and of an ovigerous female specimen (Fig. 7A, B), which accurately show complete colour tones and patterns. The photographs show that the background colour of the body is white, or white and light amber on the chelae. The shield has orange and reddish patches arranged more-or-less symmetrically on the calcified portions. The ocular peduncles are white, each with an orange-red stripe on the dorsal face, and a light orange ventral face. The antennular peduncles are semi-transparent. The antennal peduncles each have a light orange-red patch on the laterodistal face of the second segment, and an orange stripe on the lateral faces of the fourth and fifth segments; the flagella are semitransparent or light orange. The right cheliped has the chela mostly light amber with white spines or tubercles, and white patches medially; the carpus is orange dorsally, with white spines or tubercles; the merus is red dorsally, with bright white lateral and mesial faces. The colour pattern of left cheliped is similar to that of right cheliped. The ambulatory legs have dactyls mostly white except for a light orange proximally; the propodus and carpus are white except for two light orange stripes (one dorsolateral, and one ventrolateral) on the lateral face of both segments, and the mesial face of both segments are similar to the lateral face; the merus is bright white except for a dorsolateral red stripe broadening distally near the distal margin; the ischium is white with a dorsolateral light orange stripe. The pleon is orange or reddish. The uropods and telson are white with light orange.

####### General distribution.

Reported from the Southern hemisphere from 22°S to 57°S. Depth: 91–1995 m.

As discussed by Lemaitre (2004b), *Eupagurus modicellus* Stebbing, 1914 from Ascencion Island, was believed by Manning and Chace (1990) to represent *S. dimorphus*. However, Stebbing’s taxon was based on a juvenile specimen that likely does not represent *S. dimorphus*. Thus, the presence of this species as far north as Ascencion Island in the South Atlantic is considered questionable.

####### South African distribution

**Figure 8.**
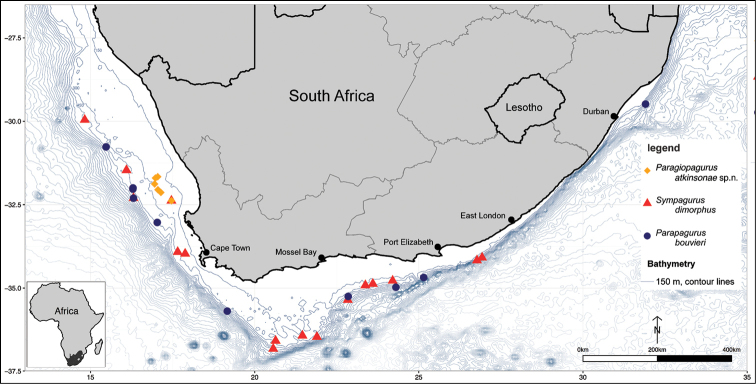
South African distribution of three parapagurid species based on specimens found during DAFF demersal research surveys.

**(Fig. 8).** Highly abundant all along the west coast, common on and along the shelf of the Agulhas Bank on the south coast, extending to offshore areas of East London; depth range of material in this study is 170–585 m.

####### Common names.

“Monkey-nut hermit crab”, “Cloaked hermit crab”.

####### Habitat.

Found living in gastropod shells, usually with actinian or zoanthid polyp attached to the shell, or in carcinoecia formed by colonies of *Epizoanthus* sp.; young have been found in scaphopod shells (Lemaitre 2004b). On the south coast of South Africa, commonly found in gastropod shells that are not covered by anthozoan symbionts.

####### Genetic data.

Sta D0520-4071, S36°27.78', E21°53.58', 401 m, male 13.2 mm, BOLD: SEAKY876-15 (MB-A066841). Sta D0520-4071, S36°27.78', E21°53.58', 401 m, male 14.6 mm, BOLD: SEAKY962-15 (MB-A066839).

##### Genus *Parapagurus* Smith, 1879

###### Parapagurus bouvieri

Taxon classificationAnimaliaDecapodaParapaguridae

Stebbing, 1910

Figs 7C, D , 8

Parapagurus bouvieri Stebbing, 1910: 357, pl. 17 (Crustacea pl. 43) (type locality: South Africa, SS “Pieter Faure”, sta 153, Buffalo River, NW 1/2W, 19 miles, 549 m)

####### Material examined.

*South Africa, West Coast*. WCDSS2012, AFR279: 2 females 8.6, 11.0 mm, sta A32144-4116, S32°18.26', E16°18.529', 369 m, 11 Jan 2012 (SAMC MB-A066800); 3 males 10.4–13.1 mm, sta A32147–5079, S32°01.87', E16°17.43', 458 m, 11 Jan 2012 (SAMC MB-A066799). WCDSS2015, AND004: 8 males 7.5–14.3 mm, 3 ovig. females 9.2–11.8 mm, 1 female 8.9 mm, sta C0372–5140, S35°41.28', E19°09.82', 551 m, 11 Feb 2015 (SAMC MB-A066793); 1 female 9.4 mm, sta C0407–5104, S33°01.92', E17°01.98', 436 m, 21 Feb 2015 (SAMC MB-A066795); 2 males 6.1–12.5 mm, sta C0420–5078, S31°59.88', E16°17.64', 812 m, 27 Feb 2015 (SAMC MB-A066796). WCDSS2016, CCH008: 1 ovig. female 10.0 mm (SAMC MB-A066429), 1 male 14.0 mm (SAMC MB-A066430), 1 ovig. female 9.3 mm (SAMC MB-A066431), 1 male 12.2 mm (SAMC MB-A066432), 1 ovig. female 10.0 mm (SAMC MB-A066433), sta D00716, S30°46.14', E15°28.44', 387 m, 9 Mar 2016.

*South Africa, South Coast.* SCDSA 2015, AND005: 1 male 11.8, sta D00570–6628, S34°40.95', E25°09.15', 556 m, 3 May 2015 (SAMC MB-A066797); 2 males 10.0–12.7 mm, sta D00573–6592, S34°58.42', E24°18.37', 758 m, 4 May 2015 (SAMC MB-A066798). SCDSS2016, AFR289: 2 males 7.4–10.6 mm, sta A32823–96971, S35°14.95', E22°50.80', 511 m, 5 Oct 2016 (SAMC MB-A066794).

*South Africa, East Coast.* African Coelacanth Ecosystem Programme (no cruise name): 4 males 7.9–12.2 mm, sta ACEP 3–6, S29°29.10', E31°54.36', 563–569 m, 20 Mar 2010 (ZRC 2013.0548).

####### Diagnosis, taxonomy.

See Lemaitre (1990; 1999; 2000), and Poore (2004).

**Colour (in life; Fig. 7C, D).** Until now, colour information on this species has been based only on Barnard’s (1950: 451, as *Parapagurus pilosimanus*) description of specimens from South Africa, and a photograph by Poore (2004, pl. 17b) only partially showing the body of a live specimen in a zoanthid carcinoecia. Herein, we describe in detail for the first time the colouration of this species, and present colour photographs of the entire body of a fresh specimen removed from it’s housing and with it’s anthozoan housing (Fig. 7C, D). Shield light orange with small white patches on posterior half, and white near anterior margin. Ocular peduncles white dorsally, turning light orange on lateral faces; corneas black to dark brown. Antennules white with light orange flagella. Antennal penduncles white except for orange lateral faces of second segments, and orange acicles; flagellum light orange except for white basal portion. Colour of chelipeds hidden by dense light brown setation, surfaces white except for some orange tint distally on fingers. Ambulatory legs white with broad orange stripe on lateral faces of meri, carpi, and propodi; dactyls orange distally and with narrow orange stripe on lateral face; weakly calcified region on lateral face of meri brownish.

####### General distribution.

Southeastern Atlantic, from Angola to South Africa, and southwestern Indian Ocean to KwaZulu-Natal (South Africa); western Pacific, from off the southern and southeastern coast of Australia, from the South Australian Bight and Queensland (Lemaitre 1999; Poore 2004). Depth: 247–990 m.

####### South African distribution

**(Fig. 8).** Highly abundant on the west coast, common on the shelf of the Agulhas Bank on the south coast, and extending to the east coast off KwaZulu-Natal; depth range of material in this study from 369–812 m.

####### Common name.

“Hairy-clawed hermit crab”.

####### Habitat.

With extremely rare exceptions, exclusively found living in carcinoecia formed by zoanthids, probably *Epizoanthus* species.

####### Genetic data.

Sta ACEP 3–6, S29°29.10', E31°54.36', 563–569 m, male 12.2 mm, BOLD: SEAKY1174–17 (ZCR 2013.0548–2). Sta D00716, S30°46.14', E15°28.44', 387 m, male 12.2 mm, BOLD: SEAKY1169–17 (MB-A066432); ovig. female 10.0 mm, BOLD: SEAKY1167–17 (MB-A066429); ovig. female 9.3 mm, BOLD: SEAKY1168-17 (MB-A066431).

####### Remarks.

As pointed out by Lemaitre (1990; 1999; 2000), this species is unique among species of *Parapagurus* in several characters. In *P. bouvieri* the corneas are weakly dilated, and the overall length of the ocular peduncles are atypically long, being distinctly more than half the length of the shield, whereas in other species of the genus the corneas are reduced, not wider than the distal width of the ocular peduncles, and the ocular peduncles are short, less than half the length of the shield. The most striking and distinctive character of this species is the presence of a weakly-calcified area on the lateral and mesial faces of the propodi of the ambulatory legs. In live specimens this area is brownish in colour (Fig. 7C), and that tone is similarly retained even in specimens that have been preserved for a long time.

## Discussion

*Sympagurus dimorphus* and *Paragiopagurus atkinsonae* sp. n. are superficially similar and could be confused if the morphology is not carefully examined. Given the scarcity of taxonomic information on South African parapagurids, it is therefore not surprising that until now the latter new species has been confounded with the former. In addition, the two species co-exist and are trawled in large numbers from the same benthic environments, and both species utilize a similar housing strategy for protection, i.e., a carcinoecia formed by potentially the same species of zoanthid polyps (in South Africa, *S. dimorphus* is also often found inhabiting shells). Morphologically, both species exhibit a marked sexual dimorphism that is expressed most visibly in males by having a long and often slender right cheliped, whereas in females the right cheliped is stout, with a broad, operculate chela. The variations in males and females of *S. dimorphus* have been documented in detail by Lemaitre (1989; 1996; 2004b). The general tone of the colouration of *S. dimorphus* and *P. atkinsonae* sp. n. is also superficially similar, i.e., white in background with orange or red stripes. However, that is where the similarity ends, as the two species differ in fundamental generic characters as defined by Lemaitre (2004b) for *Sympagurus*, and Lemaitre (2013) for *Paragiopagurus*. Most significantly, species of *Sympagurus* are the only among parapagurids that posses a vestigial pleurobranch on each side of the eighth thoracic somite, above each fifth pereopod (see Lemaitre 2004b: 89, fig. 2). Furthermore, in *S. dimorphus* the gills are quadriserial, deeply divided, whereas in *P. atkinsonae* sp. n. the gills are at most weakly divided distally. In addition, the condition of pleopods in males of these two species differ drastically, the males in *S. dimorphus* having well developed, paired first and second pleopods modified as gonopods, whereas the males in *P. atkinsonae* sp. n., lack first pleopods and only have unpaired left second pleopods. Aside from fundamental generic differences, however, in the field these two species can be best identified by differences in colouration pattern (Figs 6A–D, 7A, B). In *P. atkinsonae* sp. n. the corneas are greenish (Fig. 6C), whereas in *S. dimorphus* they are dark brown or black; the general background colouration is more orange, whereas in *S. dimorphus* it is mostly white; the chelipeds are almost entirely orange with white tubercles or spines, whereas in *S. dimorphus* most of the chelae and lateral faces of carpi are white or light amber, and the meri are bright white except for a red dorsal face; the carpi, propodi and dactyls of the ambulatory legs have three orange stripes on a light orange background, whereas in *S. dimorphus* those segments have two orange stripes on often bright white background; the meri have a large white patch on the proximal half of the lateral face and a dark orange dorsal margin, whereas in *S. dimorphus* the lateral face of the merus is almost entirely bright white, except for a red-orange stripe dorsodistally.

Even without sufficient familiarity with the other taxonomic characters that define species of parapagurids, *P. bouvieri* can also be easily separated from the other two most commonly co-occurring parapagurid species encountered in the South Africa demersal surveys, by the relative length of the antennal peduncles (peduncle and acicle distinctly exceeding distal margins of the corneas), the more slender, longer, and dorsally unarmed meri and carpi of the ambulatory legs, and shape (stout propodus and short dactyl) and armature of propodus and propodal rasp (with conical scales) of the fourth pereopod, all of which can be easily compared in the publications cited herein for each of the three parapagurid species encountered in the demersal surveys. Compared to *P. atkinsonae* sp. n. and *S. dimorphus*, *P. bouvieri* inhabits different carcinoecia formed by different zoanthid species. Whereas the carcinoecia is firm, rigid, stabilized by imbedded grains of sand, and usually dark brown in the former two species, the carcinoecia inhabited by *P. bouvieri* is softer and gelatinous, grey to rosy in colour and almost neutrally buoyant in sea water. Additionally, the colour photographs (Figs 4C, D, 6A–C, 7A–D) presented for *P. bouvieri*, *Sympagurus dimorphus*, and *Paragiopagurus atkinsonae* sp. n., complete the set of morphological comparisons that should enable biologists to identify these three species.

Despite the considerable sampling effort along the entire extent of the South African offshore demersal survey grounds, *P. atkinsonae* sp. n. was confined to a small area on the West Coast, where it appears to be common. The distribution, being restricted to an area of only 43 nautical miles in the north-south, and only 25 nautical miles in the east-west direction, is unusual for any parapagurid species, which normally have wide-spread distributions (e.g. Lemaitre 1999; 2004b; 2013; 2014)). For example, in the South African benthic abundance surveys, the distributions of *S. dimorphus* and *P. bouvieri* extend from the westernmost fishing grounds from the Namibian boarder to the easternmost sites west of Port Elisabeth. Using the newly provided identification information, future studies should monitor the occurrence of *P. atkinsonae* sp. n. in the demersal abundance surveys. Should it be confirmed that *P. atkinsonae* occurs exclusively in the small area of the South African West Coast then this area should be given more research attention. The area does not obviously appear oceanographically or biologically distinct, but more detailed sampling of the benthic invertebrate community and ecosystem might reveal that it provides specific habitat conditions that could be important to both study and protect from future anthropogenic impacts.

## Supplementary Material

XML Treatment for Paragiopagurus atkinsonae

XML Treatment for Sympagurus dimorphus

XML Treatment for Parapagurus bouvieri
